# Piloting community-based medical care for survivors of sexual assault in conflict-affected Karen State of eastern Burma

**DOI:** 10.1186/1752-1505-7-12

**Published:** 2013-05-21

**Authors:** Mihoko Tanabe, Keely Robinson, Catherine I Lee, Jen A Leigh, Eh May Htoo, Naw Integer, Sandra K Krause

**Affiliations:** 1 Women’s Refugee Commission, 122 East 42nd Street 11F, New York, NY 10168-1289, USA; 2 Global Health Access Program, Mae Sot, Thailand; 3 Burma Medical Association, Mae Sot, Thailand; 4 Karen Department of Health and Welfare, Mae Sot, Thailand

**Keywords:** Burma, Sexual assault, Reproductive health, Community-based medical care, Internally displaced persons, Community health workers, Conflict setting

## Abstract

**Background:**

Given the challenges to ensuring facility-based care in conflict settings, the Women’s Refugee Commission and partners have been pursuing a community-based approach to providing medical care to survivors of sexual assault in Karen State, eastern Burma. This new model translates the 2004 World Health Organization’s *Clinical Management of Rape Survivors* facility-based protocol to the community level through empowering community health workers to provide post-rape care. The aim of this innovative study is to examine the safety and feasibility of community-based medical care for survivors of sexual assault to contribute to building an evidence base on alternative models of care in humanitarian settings.

**Methods:**

A process evaluation was implemented from July-October 2011 to gather qualitative feedback from trained community health workers, traditional birth attendants, and community members. Two focus group discussions were conducted among the highest cadre health care workers from the pilot and non-pilot sites. In Karen State, eight focus group discussions were convened among traditional birth attendants and 10 among women and men of reproductive age.

**Results:**

Qualitative feedback contributed to an understanding of the model’s feasibility. Pilot site community health workers showed interest in providing community-based care for survivors of sexual assault. Traditional birth attendants attested to the importance of making this care available. Community health workers were deeply aware of the need to maintain confidentiality and offer compassionate care. They did not raise safety as an excess concern in the provision of treatment.

**Conclusions:**

Data speak to the promising “feasibility” of community-based post-rape care. More time, awareness-raising, and a larger catchment population are necessary to answer the safety perspective. The pilot is an attempt to translate facility-based protocol to the community level to offer solutions for settings where traditional methods of post-rape care are not accessible for women and girls that need it most.

## Main text

Women and girls in conflict and other displacement settings are at increased risk of sexual violence [1]. This type of violence increases the risk of unwanted pregnancy, unsafe abortion, and sexually transmitted infections (STIs), including HIV [2]. Despite their increased vulnerability, care for those who have survived sexual violence is limited in humanitarian settings, as service providers are often ill-equipped to treat survivors, and facilities may lack supplies and trained providers at the height of insecurity [3,4]. Distance to a health facility and stigma associated with sexual violence are also barriers to accessing care [5].

## Background

The minimum health response for sexual assault is a facility-based intervention and is outlined in the World Health Organization’s (WHO) 2004 *Clinical Management of Rape Survivors: Developing protocols for use with refugees and internally displaced persons*[6] (see Table 1).

**Table 1:** **WHO*Clinical management of rape survivors*protocol**

	
Facility-based minimum response (WHO, 2004)	Collecting minimum forensic evidence with the survivor’s consent if capacity exists for its use.
	Conducting a minimum medical examination with the survivor’s consent.
	Providing compassionate and confidential treatment that includes:
	Treatment and referral for life-threatening complications
	Treatment or preventive treatment for sexually transmitted infections (STIs)
	Emergency contraception to reduce the risk of pregnancy
	Care of wounds
	Supportive counseling
	Referral to social support and psychosocial counseling services
	Comprehensive treatment includes the provision of:
	Post-exposure prophylaxis (PEP) to reduce the risk of HIV transmission
	Tetanus toxoid/Tetanus immunoglobin to prevent tetanus
	Vaccines to prevent hepatitis B

An extensive literature review conducted in 2008 revealed that no research had been conducted in development or relief settings on community-based delivery of medical care for survivors of sexual assault where community health workers (CHWs) provide direct services, although a project in the Democratic Republic of the Congo (DRC) has offered physician- and nurse-staffed mobile health services and has engaged local community members to encourage health-seeking behavior around sexual assault [7]. When the components of WHO’s minimum package of care are broken down, existing research from various settings and guidance show that emergency contraception [8-11]; antibiotics, including for preventive treatment of STIs [11-13]; and the other interventions [11,14-16] can be provided safely at the community level. Indeed, the Population Council showed the effective police distribution of emergency contraception to sexual assault survivors in Zambia [17]. Research is also available from development contexts on community-based delivery of tetanus toxoid [11,18,19] and hepatitis B [20] vaccines through the safe and proper use of the Uniject device, both of which have received WHO prequalification [21]. While guidelines on post-exposure prophylaxis (PEP) allow for the dispensing of the full 28-day course at the initial visit in emergency settings where access to health facilities is limited [22], only nurses and above are permitted to initiate the PEP regimen under WHO task-shifting guidance [23].

Given the challenges to ensuring facility-based care and the existing evidence on the safe provision of individual components of WHO’s minimum care package from development settings, this paper describes the findings from a process evaluation of a pilot project on community-based medical care for survivors of sexual assault. The examined approach is a novel model that has never before been implemented in any setting. The intent in investigating this innovative method that translates the WHO facility-based protocol to the community level is to advance research on alternative models of care and contribute to building an evidence base that would demonstrate whether such a method is safe and feasible in humanitarian settings.

### Burma background

For over five decades, ongoing conflict and military misrule in Burma’s ethnic minority border regions have destroyed the fabric of civil society, impoverished and uprooted civilians and their communities, and damaged health and education systems [24]. As a result, communities experience low health indicators, including for reproductive health (RH) [25]. Karen State in eastern Burma has been an area of active conflict, housing approximately 100,000 internally displaced persons (IDPs) [26]. Militarization threatens the IDPs, with patrols and landmines among the most significant dangers, and forced labor and movement restrictions affecting livelihoods [27]. Intensified conflict in Karen State in 2009 resulted in more than 4,000 Karen villagers fleeing to Thailand [28].

United Nations (UN) reports have described the trend of sexual violence in eastern Burma as “particularly alarming” [29], with the UN Special Rapporteurs on Violence against Women and on Torture and Other Cruel, Inhuman or Degrading Treatment or Punishment discussing the state of impunity, citing case examples from Karen State [30,31]. Local reports from community-based organizations focusing on rape perpetrated by soldiers in Karen State and other locations in eastern Burma cite sexual assault as a primary concern during displacement [32-34]. While data on the prevalence of sexual violence is not available for Karen State, research published in 2011 on the results of a population-based assessment using multistage household cluster sampling in Chin State, western Burma, found that 2.8% of households reported rape/sexual violence in a 12-month period [35].

The Global Health Access Program (GHAP), Burma Medical Association (BMA), and the Karen Department of Health and Welfare (KDHW) have operated community-based programs to address the health needs of IDPs in eastern Burma where international agencies have had limited to no access [36]. From 2005 to 2008, GHAP, BMA, and The Johns Hopkins Center for Public Health and Human Rights implemented the Mobile Obstetric Maternal Health Workers (MOM) Project, a task-sharing, tiered initiative to address the maternal health needs of nearly 46,000 IDPs in four states, including Karen State. The initiative provided mobile services for antenatal care, clean delivery, postnatal care, and basic emergency obstetric care to communities [25,37]. KDHW has implemented an adapted program since 2007, providing essential health services to 32,500 IDPs [38].

### Pilot project to provide community-based medical care to survivors of sexual assault

Given the difficulties of ensuring facility-based care in crisis-affected settings, the Women’s Refugee Commission partnered with GHAP, BMA, and KDHW in November 2009 to pilot a community-based model to provide medical care to survivors of sexual assault as an option of care in situations with barriers to facility-based care. Medical care for survivors of sexual assault had not been previously available in BMA and KDHW programs; the closest health facility providing such services was Mae Tao Clinic in Mae Sot, which is a several days’ walk across the Thai-Burmese border and through the Thai government’s border security controls. Recognizing these obstacles, providing referrals to Mae Tao Clinic was unrealistic, and establishing standing health facilities risked sabotage from the Burmese military’s deliberate tactics to destroy health infrastructure [24].

The study, therefore, aimed to explore whether community-based medical care for survivors of sexual assault is safe and feasible in this humanitarian setting and the challenges of providing care in this manner. Safety pertained to any additional risks for survivors and CHWs as post-rape care is made available at the community level, and the ability of CHWs to provide care and address any consequences according to established medical protocol. Feasibility was related to CHW and community acceptance of addressing gender-based violence (GBV) and providing care around this sensitive issue; CHWs’ ability to provide confidential care as a package of care traditionally offered by higher cadre health workers; and whether logistics and secure information management systems could be sustained for this effort. Through an in-depth analysis of practical, legal, and ethical considerations, the pilot built upon BMA and KDHW’s existing logistics framework, cadre of CHWs, community rapport, and monitoring mechanisms for community-based delivery of post-rape care. As the CHWs were experienced in administering individual components of the package of care, the pilot tasks complemented the scope of services already provided by the trained CHWs.

#### Site selection

For ethical reasons, the four selected locations among BMA and KDHW’s sites met the following two conditions: 1) no other care for survivors was available; and 2) the site mirrored a situation of insecurity where access to care posed severe challenges (see Table 2). The sites served as proxies for heightened emergency situations where insecurity leads to dysfunctional medical logistics, shortage of health staff, and a lack of safe access to health facilities. The relative stability of the selected sites allowed for a consistent supply chain, monitoring visits by staff, data collection, and community education activities on issues of gender and violence. Moreover, the literacy and skills levels of the highest cadre of CHWs justified the addition of a new package of services.

**Table 2:** Pilot project sites

**Site**	**Population**	**Distance from nearest hospital providing medical care for sexual assault survivors**
Burma Medical Association MOM Project		
Site 1	4,094	3 days walking distance
Site 2	3,536	1 day walking distance
**Total**	**7,630**	
Karen Department of Health and Welfare RH Program		
Site 3	2,192	2 days walking distance
Site 4	1,827	2 days walking distance
**Total**	**4,019**	

#### Target population

As no specific prevalence rate for sexual assault was available in the selected sites or Karen State, the pilot assumed 25% of the population to be women of reproductive age, and that 2% would experience sexual assault per *Reproductive Health in Humanitarian Settings: An Inter-agency Field Manual*[2]. Hence, it was projected that 58 women would be sexually assaulted annually in the four sites combined. Taking into account gross under-reporting [24], the number of actual cases coming forth for care was expected to be low. While the WHO protocol extends to children, neither children nor men were directly included in the pilot scope. CHWs would not decline care; yet men, especially, were not expected to seek services, due to cultural sensitivities around sexual assault, as was found during in-depth interviews with key informants and focus group discussions (FGDs) organized among CHWs at the time of pilot start-up. Cases among young children were also perceived to be relatively few; the pilot focused on women and adolescent girls until CHWs could demonstrate required competencies in their new roles.

#### Scope of cases to be treated

The pilot defined “sexual assault” as any act of “forced sex,” including non-marital rape, marital rape, and other acts of coerced sexual intercourse. While a varied understanding of terms was expected among health staff and community members, CHWs were trained to treat survivors that requested care for any act of sexual assault. CHWs did not routinely screen for sexual assault in the provision of primary health care and other RH services, but were instructed to provide the minimum package to those who requested and consented to receive treatment.

#### Scope of treatment to be provided

The pilot focused on the provision of eight activities (see Table 3). Comprehensive components of the WHO protocol were excluded, including the collection of forensic evidence, dispensing of PEP, and the provision of tetanus toxoid and hepatitis B vaccines. Prosecuting for rape in the IDP areas of Burma is not yet feasible [39], and, as identified in the pre-pilot FGDs, communities rely on traditional means of adjudication for sexual violence. In light of the need for judicial or other capacity to warrant the collection of forensic evidence [6], and safety and security concerns for both survivors and CHWs, forensic evidence was not to be collected. Due to low HIV prevalence in the Karen communities (an estimated 0.7% for Burma as a whole [40]) and the current WHO task-shifting policy on PEP initiation, PEP was also excluded. This was consistent with Mae Tao Clinic’s policy, where PEP is offered on a case-by-case basis to sexual assault survivors. Additionally, as HIV testing is only available at BMA’s sites and is used solely for screening blood for transfusions, this element was not included in the model’s protocol [38]. The two vaccines were additionally eliminated as a result of difficulties in sustaining a cold chain and follow-up activities for the hepatitis B vaccine.

**Table 3:** Pilot project activities

	
Activities implemented in the pilot project	Conducting a medical examination with the survivor’s consent
	Providing compassionate and confidential treatment that includes:
	Treatment and referral for life-threatening complications
	Treatment or preventive treatment for STIs
	Emergency contraception to reduce the risk of pregnancy
	Care of wounds
	Supportive counseling
	Basic psychosocial care
	Referral to mobile facilities as available

In terms of referrals and the lack of permanent facilities, interventions from the pilot sites were limited to trauma treatable at the mobile clinic level. While international guidelines on GBV stipulate a multi-sectoral approach to GBV programming [41], the realities in Karen State make it impossible to ensure police, judicial, protection, and other measures [42]. The pilot was therefore limited to the provision of medical services and basic psychosocial care, drug regimens of which adhered to the 2007 *Burma Border Guidelines*[43].

#### Training

The initial five-day training on care for survivors of sexual assault for the highest cadre of CHWs in the tiered system was held at Mae Tao Clinic, as part of the cohort’s semi-annual training. These highest cadre CHWs have been trained to provide RH services, including basic emergency obstetric care. Two workers from each of the four sites were trained by Mae Tao Clinic and GHAP staff and attended a three-day refresher training every six months. The curriculum was based on WHO’s 2004 protocol, with additional modules to address listening, counseling, referral, confidentiality, violence, gender, and community responses to such issues. The training included lectures and hands-on demonstrations. Sessions on confidentiality built on the CHWs’ prior experience in family planning counseling and HIV testing for safe blood transfusion.

Once back in their villages, the highest cadre workers trained groups of traditional birth attendants (TBAs), with group sizes ranging from 10 to 23, on gender, GBV, and care for survivors. TBAs served as conduits to raise awareness within the community, and in the event survivors reported, to inform the higher-level workers for the immediate provision of care.

#### Routine data collection and information management

In their routine work, CHWs complete forms during consultations, which they retain at the field level. They compile monthly report forms, which they send to Mae Sot through secure channels. The pilot project built on this existing system, further taking into account global GBV data collection and management efforts, including the International Rescue Committee/UN Population Fund (UNFPA)/UN High Commissioner for Refugees’ *GBV Information Management System* (GBV IMS) project [44]. The intake form and the information management protocol from the GBV IMS were adapted to focus exclusively on the treatment provided. In view of security risks, the adapted intake form, “Form A,” does not note the location, identifier, or perpetrator information. Such information was thought to put both the survivor and treating CHW at risk, especially if the perpetrator was a military personnel and could retaliate if the case became known. CHWs complete Form A at the time of initial consultation and again two weeks later, as follow-up to any care received. Duplicate copies are sent with other routine data on a quarterly basis to Mae Sot, where they are stored in a locked cabinet at the central office for review by pre-assigned staff for data analysis.

WHO’s guidelines note the right of survivors to obtain a medical certificate for proof of care received [6]. As CHWs and the community at large are aware of potential risks to their physical security—due to the circumstances of their displacement—requests for documentation were to be addressed on a case-by-case basis. The CHWs would only provide records to survivors themselves, unless they were to the parent or guardian of a minor, per standard GBV guidelines [45].

#### Ethical considerations

As this was the first attempt to translate the WHO facility-based protocol to the community level, the Women’s Refugee Commission and partners took utmost care in ensuring ethical standards. In close consultation with the Inter-agency Working Group (IAWG) on Reproductive Health in Crises New Technologies Working Group, the Women’s Refugee Commission reviewed various guidelines on sexual violence data collection, including WHO’s 2007 *Ethical and Safety Recommendations for Researching, Documenting and Monitoring Sexual Violence in Emergencies* and WHO/UNFPA/UNICEF/UN Action’s 2008 *Sexual Violence in Conflict: Data and Data Collection Methodologies* prior to pilot initiation [45,46].

Health care providers were instructed to seek consent to treat and to inform survivors of the confidential nature of the treatment provided. None of the sites were required to report cases of sexual assault to traditional judicial systems. At the field level, the highest cadre of workers ensured the safety of the intake forms, and emphasis was placed on not recording unique identifier information. The provision of care focused on the highest cadres until evidence could be established on the safety and feasibility of the approach, whereupon lower levels could be empowered to directly provide care.

While the project was designed to address security concerns, BMA and KDHW further developed contingency plans to address potential negative consequences as a result of community-based care, such as scenarios for what to do if a perpetrator discovers that a survivor has sought assistance. The overall pilot received approval from the local ethics review board comprising the Mae Tao Clinic and the ethnic health departments.

## Methods

From July to October 2011, the Women’s Refugee Commission and GHAP conducted a process evaluation of the pilot project. FGDs were undertaken in Mae Sot in July among half of the highest cadre CHWs from the pilot sites to assess their perspectives and experiences regarding the approach, examine their comfort levels in providing this care, and identify any challenges to maintaining confidentiality. Additional FGDs among nine non-pilot CHWs were also conducted in July to examine their perspectives. Several non-pilot CHWs had been trained in medical care for survivors of sexual assault along with the pilot site CHWs, but had not been expected to initiate services until implementation could be assessed in the pilot sites.

In the interest of time and available resources, onsite translation was conducted, whereby the FGDs were led by GHAP staff who facilitated the discussion in English. Mae Tao Clinic staff translated both the questions and responses from English to Burmese and vice versa, and a note-taker took notes in English. Verbal consent was obtained for participation. The sessions were not audio-recorded, as some participants expressed reluctance. After each session, the facilitator, translator, and note-taker debriefed to review the discussion for a better understanding of nuances [47] and type preliminary notes.

TBAs and community members in the pilot sites participated in FGDs facilitated by the pilot CHWs who had received a two-day training in FGD methodology. The intention of the field-based FGDs was to solicit TBA perspectives and experiences on the approach, including on the issue of confidentiality, and examine factors that would affect the community’s willingness to seek health care after sexual assault. For ethical and safety reasons, questions focused on the approach and not on individuals’ experiences of sexual assault. Similar to the Mae Sot FGDs, the Women’s Refugee Commission and GHAP developed the FGD tool, with input from BMA and KDHW. The FGD tool was translated into Burmese and Karen by BMA staff and reviewed by the Mae Tao Clinic translator. During the training, the CHWs reviewed all questions and facilitated mock discussions to ensure accuracy of the translations.

Once back in Karen State, the trained CHWs implemented eight FGDs among TBAs and 10 FGDs among women and men of reproductive age in the four communities in September-October 2011. Participants ranged from six to 11 people in each group. Taking into consideration security risks, no FGDs were held in locations of active conflict where gathering persons for any purpose could have been misinterpreted to put facilitators and participants at risk. The CHWs facilitated the discussions in the Karen language. Four TBA FGDs and six community FGDs were taped. Audio recordings were made only if participants were comfortable and all participants provided verbal consent. The tapes and notes were securely transported to Mae Sot in November 2011 for transcription and translated into English by BMA and KDHW staff. All recordings and original notes were destroyed once the analysis was completed.

Analysis of the FGD data from the CHWs, TBAs, and community members was conducted separately. The Mae Sot CHW data were analyzed using the open source qualitative data analysis software TAMS. Sixteen tags were used that included: “understanding GBV,” “confidentiality,”“comfort with GBV,” and “clinical care for sexual assault.” The field-based FGDs were analyzed through grouping findings under common themes, similar to those of the Mae Sot data, as well as performing key word searches to determine trends and to identify aberrant cases. Monitored activities, such as key project indicators, including pre-/post-test scores of trained CHWs and TBAs, and components of medical care provided, were also reviewed.

## Results

### Pilot and non-pilot health care worker FGDs (Mae Sot)

Overall, the data revealed the pilot site CHWs to be comfortable with the topic of GBV, including sexual assault, and knowledgeable about clinical skills and needs of survivors (see Table 4). Although no cases of sexual assault were reported to or treated by the CHWs, the pilot site CHWs demonstrated comfort with the subject of sexual assault and good understanding of medical treatment. The two skills about which they reported lacking confidence were history-taking and psychosocial care. The pilot site CHWs found asking questions about sexual assault somewhat uncomfortable, with one quoted as saying, “History-taking is challenging because the case history questions are difficult to ask and can make the patient uncomfortable. The woman could feel shy and we also feel uncomfortable asking, too. We have not yet had to ask, however; we only suspect that we will hesitate to ask.”

**Table 4:** Summary of key findings from focus group discussions

**Participants**	**Key findings**
Pilot site CHWs	Comfortable with topic of GBV, including sexual assault.
	Knowledgeable about clinical skills for survivors of sexual assault.
	Less confident in history-taking and psychosocial care.
	Understood meaning of confidentiality, use of forms, and information management processes.
	Security not seen as an excess concern.
	Recognized more time is needed to train TBAs.
	Recognized more time and awareness-raising are needed to encourage survivors to seek care.
	Reported domestic violence as the most common type of GBV in the community.
	Noted no reported cases or other issues to suspect sexual assault in the community.
Non-pilot site CHWs	Interested in providing treatment for sexual assault survivors.
	Showed some confusion about definition of sexual assault and their role in caring for survivors.
	Reported domestic violence as the most common type of GBV in the community.
	Noted no reported cases or other issues to suspect sexual assault in the community.
TBAs	Understood role as providers of encouragement and referrals.
	Need to maintain confidentiality was not reported as a major challenge, although understanding of confidentiality was mixed.
	Showed mixed feelings regarding safety in assisting survivors.
	Shared interest in learning more about GBV and how to help the community.
	Reported domestic violence as the most common type of GBV in the community.
Community members	Shared primary barriers and challenges for survivors to accessing care as shyness; fear of others’ opinions; shame; and concerns that they may not receive help.
	Agreed trusted persons in the community exist from whom survivors may seek care.
	Suggested the community needs to feel comfortable in seeking care from a CHW or TBA.

The pilot CHWs also demonstrated full understanding of confidentiality, use of forms, and the process and means by which collected information would flow from their sites to Mae Sot. They accurately recited the information flow channels, and how the forms would be stored. They further underscored the importance of confidentiality through noting, “To encourage people to come for care, we will emphasize the concept of confidentiality, assuring people that their case will be kept private.”

Security was not seen as an excess concern for the pilot site CHWs, as they perceived their risks of providing care to survivors to be the same as if they were providing any other care or treatment. The common understanding was, “We feel safe. We would feel nervous treating the first patient, but only because we do not yet have experience. If someone came to us with this problem, we would feel safe and treat them as best we could.”

Regarding the engagement of TBAs, the CHWs believed that although they had each conducted training sessions with TBAs in their villages, it was difficult to gauge the TBAs’ level of interest or understanding of the topic. One reported that a TBA in her village resented being trained on the components of medical care and survivor referrals, saying that her job already had too many responsibilities, so “don’t make me busy.” The pilot site CHWs agreed they would need more time to properly train the TBAs and to organize community education sessions to encourage survivors to come for care. One worker proposed that “three or four community meetings may help to foster change. If community education meetings are held once every three months over the next year, the community will be prepared to discuss these issues and begin seeking care.”

The non-pilot site CHWs showed interest in the provision of treatment for sexual assault survivors. The FGDs uncovered more confusion among them, compared to the pilot site workers, about the definition of sexual assault and their role in caring for survivors. One non-pilot CHW described “collection of evidence” from a sexual assault survivor as part of a CHW’s role, even though legal justice is not available in Karen State and this task had hence been excluded from the pilot scope.

No cases of sexual assault were seen or heard of by any of the CHWs during the study period, and there were also no reported cases of STIs that made them suspect sexual assault. There was general consensus among all CHWs that domestic violence was the most common type of GBV in their communities. Reports of sexual assault perpetrated by military forces were rare, and rumors of other attacks were of incidents that had occurred in migrant areas. Hence, while the CHWs did not suspect missed cases, the pilot site CHWs agreed that to encourage survivors, in addition to confidentiality, “We also want to remind the community that we are capable to provide this care and have been trained to do so as health workers.”

### Traditional birth attendants (Karen State)

An overall review of the data demonstrated that in cases of sexual assault, TBAs showed a good understanding of their own role. They agreed that “the role is to give them encouragement, to give them suggestions and send them to clinic (CHWs) for treatment.” Others noted, “Because we are TBAs we need to take responsibility to take care of the patients who have been sexually assaulted and we will help them as we can.”

As with the CHWs, the need to maintain confidentiality was not reported as a major challenge, although understanding of what confidentiality entailed was mixed. While participants in one group noted they felt “uncomfortable,” there was agreement across groups that keeping health information private “was not too hard in our work.” TBAs stated that they could ensure survivor confidentiality and safety through responses such as, “We need to keep [confidential information] in our minds for the patients.” Despite such understanding, TBAs in one FGD noted they would need to report incidents to a village leader, lawyer, advocates, or the Karen Women’s Organization (KWO). KWO was mentioned by several FGDs, suggesting that other actors could possibly be included in the confidentiality circle.

TBAs reported mixed feelings regarding safety in assisting survivors. Responses suggested that TBAs were concerned about their own safety; however, they noted their concerns would not hinder their ability to act. “Yes [we are concerned], but we help them. If we [do] not, they will face problems,” was a common response. Others simply noted, “No, I am not afraid. I…just help them.” Many TBAs reported that they would take measures to mitigate safety concerns, such as involving higher cadre workers or the village leader where appropriate.

Overall, TBAs wanted to learn more about GBV and how to help the community. Common feedback included, “We hope that you give us more knowledge and education about GBV in our community,” and “We feel happy about our ability to support those that have been sexually assaulted and we want to learn more about GBV in our work.” Several TBAs further revealed their wish to be empowered to provide treatment directly to survivors, saying, “We need [a] medic or trainer to teach us definite way [about] how to give treatment [and] which medicines we should give [to] the patients.”

TBAs viewed domestic violence as a problematic and prevalent type of GBV, corroborating the data from the CHW FDGs. Some TBA FGDs reported “soldier rape” as the most problematic, and while two groups reported seeing cases of STIs that made them suspicious of sexual assault, they all agreed that survivors’ feeling “afraid, shy, and worried” potentially hindered their ability to seek health care. In order to increase the number of survivors seeking medical care, TBAs said they would “guide them to go to the clinic (CHWs), help them, give them suggestions, and if she doesn’t want to go to clinic (CHWs), we will try to explain to her and go with her together.”

### Community members (Karen State)

Data from the community FGDs showed the limited outreach of awareness-raising activities, as only one group was aware of the GBV-related informational activities. However, feedback reflected some of the ongoing barriers that would need to be addressed for survivors to willingly seek care. The primary barriers and challenges for women and girls voiced across all groups was shyness; fear of others’ opinions, such as the scolding of parents or being looked down upon by community members; shame; and concerns that they may not receive help. A frequent remark was, “They are not telling to other people because they are shy, afraid other people look down [on] them, afraid [that] other people don’t care [about] them or help them.” Others reiterated: “Some are feeling afraid so much.”

Despite the numerous perceived barriers to reporting an assault, all groups reported that there are trusted persons in the community from whom survivors could seek medical care. These included CHWs, medics, TBAs, traditional medical personnel, and parents, with CHWs and TBAs most often cited. “If someone has been sexually assaulted, we would seek medical care from [a] health worker or TBA or traditional medical [person],” was a common response. Three groups noted that they may report incidents of sexual assault to the village leader, although it was unclear whether they themselves would report to the village leader for personal incidents, or that they would report if they heard about the incident in their community.

All FGDs suggested that the community needs to feel comfortable in seeking care from a CHW or TBA. They noted, “The community needs to feel comfortable going to a health worker or TBA to seek medical care,” and recommended that messages of encouragement should focus on, “Don’t worry, [it was] not only you [who experienced] violence. Don’t be shy and go to clinic (CHW) and get treatment. Don’t be afraid.”

## Discussion

The findings that pilot site CHWs seemed comfortable with the subject of sexual assault; demonstrated good understanding of medical treatment; showed full understanding of confidentiality, use of forms, and information channels; and did not raise security as an added concern, are promising in terms of assessing the feasibility of the community-based model. Issues of confidentiality are nonetheless noted, especially at the TBA level. While direct questions regarding confidentiality were well understood, responses on the need to report cases to the village leader, lawyer, advocates, or the KWO reflect a possible broader understanding on their part regarding the concept of confidentiality. As their role in the pilot in its current form focuses on awareness-raising and as providers of encouragement rather than the actual prescribers of treatment, the only persons they should theoretically inform are the CHWs. More TBA trainings on their roles are expected to address this; however, their mention of KWO is of significance, especially as BMA and KDHW recognize the protective role KWO plays for women, and they have begun linking with the organization to coordinate a more robust response for survivors’ care and protection.

Regarding concerns for safety as expressed by TBAs, more information is required to determine whether the perceived fears are in excess of what they experience for other health concerns. As none of the CHWs reported safety as an excess problem, TBAs may feel reassured if they are better informed that they need not disclose incidents to other stakeholders. Of further importance is that while TBAs suggested informing advocates and others as a strategy to encourage survivors to come for care, feedback from the community did not mention the involvement of other actors as helpful in survivors’ ability or impetus to seek care. Given these findings, divorcing the “legal” and health implications may offer ways to minimize security risks and further encourage survivors to seek care.

Accounting for the limited number of awareness-raising sessions on gender, GBV, and medical care for survivors of sexual assault in the pilot sites, it is not surprising that community members exhibited reservations on disclosing incidents to even CHWs who could offer care. Shyness, fear of others’ opinions, and worries that they may not receive help are legitimate concerns for any survivor [4].

The FGDs also revealed, however, that trusted persons indeed exist from whom survivors may wish to seek care. These persons include CHWs, medics, TBAs, and family members. The community suggested that survivors could be encouraged to come forth if they felt comfortable seeking medical care from a CHW or TBA. Additional training for CHWs and TBAs on counseling, empathy, and listening skills may help increase their trust within the community. As suggested by the pilot site CHWs, more time and awareness-raising sessions are also expected to be helpful for survivors.

In terms of answering the study question, while feasibility can be discussed, as there have been no reported cases in the four pilot sites, safety of the community-based model is yet to be determined. The lack of survivor reporting is presumably due to a combination of factors, possibly including no incidents occurring in the pilot period, the sensitivities around rape and sexual assault, and limited community awareness of available services to prevent health consequences.

Several critical and positive points can be drawn from the process evaluation, however. One is the recognition and inclusion of domestic violence as a common form of GBV/sexual assault by the health providers in the pilot setting. This perception is important, as, according to the data, some CHWs had not previously recognized domestic violence or violence in an intimate partner relationship as a form of GBV or possibly constituting sexual assault. The trainings themselves did not include a focus on intimate partner assault; yet, both the pilot and non-pilot site CHWs expressed interest in learning more about domestic violence and stated it as an important issue for community awareness. Changes in mindset, therefore, are already visible from the CHW perspective, and with time and further outreach, this attitudinal change may be reflected at the community level.

Second, both the pilot and non-pilot site CHWs expressed enthusiasm about the ability to provide medical care to survivors of sexual assault in their respective communities. The CHWs had much faith in the role of TBAs. Additionally, feedback reflecting TBAs wanting to learn more and possibly even deliver care is promising in that once safety and feasibility of community-based care can be established, potential exists for training lower cadres in the provision of treatment. This type of medical care is presumably the most intimate and accessible form of care as it most closely resembles woman-to-woman care.

The process evaluation has revealed that a further extension of this project will be highly beneficial for the study, to enable the pilot communities to become more familiar with the availability and value of care, allow CHWs to further win the trust of the communities in their ability to provide confidential care, and add additional sites to increase the population base. BMA has expressed its desire to integrate GBV awareness into its existing services, such as during family planning counseling and delivery care. KDHW has also noted its interest in introducing the approach to all of its sites and integrating it with RH, immunization, malaria, and other services. Screening can be a critical component to ensuring survivors have access to medical care [48], and hence, this is an area that can be further explored. A cold chain is now feasible in a number of the pilot settings, which provides opportunities to add tetanus toxoid and hepatitis B vaccines to the post-rape care package, allowing the pilot to explore more than minimum care.

The initiation of PEP by lower cadre health care workers is still a hindrance to completing the task-shifting of post-rape care activities to the community level. While poor adherence to PEP has been documented as problematic due to side effects, studies have recommended that the risk of low compliance should not deter health providers from offering PEP, but providers should instead improve strategies for compliance [49]. Research is also increasingly available from crisis-affected settings on the outcomes and experiences of offering HIV treatment (anti-retrovirals) in crisis-affected contexts, including from the DRC, where Médecins Sans Frontières (MSF) reported high adherence levels and clients still coming for refills, even during the height of the emergency [50]. Other experiences documented by MSF note that with commitment, simplified treatment, monitoring, and adapting for potential instability, HIV treatment can be feasibly and effectively provided in conflict settings [51]. Studies are necessary, however, to examine the safety and feasibility of CHW initiation of PEP, to facilitate discourse around policy change for current facility-based protocol to be inclusive of community-based options.

### Limitations

The primary limitation is the lack of survivor reporting. An extended pilot is necessary to determine safety as it relates to task-shifting of medical care. Further, findings can only speak to the minimum package of services per WHO guidelines.

Regarding the process evaluation, due to security and other logistical constraints, not all pilot and non-pilot CHWs were able to convene in Mae Sot for the activities. However, every effort was made to capture their perspectives through BMA and KDHW’s interactions with all CHWs.

In Karen State, as the CHWs conducted the FGDs among TBAs and community members, the latter groups may have felt hesitant to voice negative opinions about the CHWs or TBAs who are the community’s only source of health services. The FGD training for the CHWs, however, emphasized objectivity, and the CHWs further practiced how to maintain a neutral and encouraging environment.

Translation error is possible across all sessions, at the time of note-taking for the FGDs in Mae Sot, and transcription for the field-based sessions. The debriefing session in Mae Sot was intended to prevent the loss of information. As the set-up of the project in an insecure terrain had CHWs only in Mae Sot during their semi-annual trainings, some of the field data may have been lost due to inability to confer with facilitators and note-takers any possible misunderstanding of the transcribed TBA and community data.

## Conclusion

Rich data speak to the promising feasibility of community-based care for survivors of sexual assault in crisis-affected settings. More time, awareness-raising, and a larger catchment population are necessary to address the safety perspective. The lack of survivor reporting does not negate the options that community-based care can possibly offer, especially as barriers to care in crisis settings are well known and new approaches are warranted to enhance access for survivors [4]. The enthusiasm of BMA and KDHW staff to integrate care for survivors across their programs is a testament of the utility and promises of this community-based approach in settings where insecurity and other barriers prevent access to facility-based care.

Evidence surrounding community-based approaches is aimed at contributing to global commitments to providing medical and psychosocial support to survivors of sexual assault in conflicts, the urgency of which has been recognized in UN Security Council Resolutions 1325, 1820, 1888, 1889, and 1960 on Women, Peace, and Security [52]. As the global community focuses on monitoring and reporting efforts of sexual violence perpetrated in conflict, the need to ensure service availability is paramount. This alternative approach to facility-based care may offer solutions to settings where traditional methods of medical care are not practical for women and girls that need it most.

## Competing interests

The authors declare that they have no competing interests.

## Authors’ contributions

MT and SKK conceptualized and designed the overall study, with input from CIL. CIL, JAL, EMH, and NI were responsible for pilot implementation at the field level. MT, KR, JL, EMH, and NI developed the process evaluation methodology and tools, and took part in data collection efforts. MT and KM performed the data analysis. MT conceptualized the paper and was principal author; KR, CIL, and SKK contributed to the writing process. All authors reviewed and approved the final text.
